# Clinical and laboratory associations of mannose-binding lectin in 219 adults with IgG subclass deficiency

**DOI:** 10.1186/s12865-019-0296-x

**Published:** 2019-05-22

**Authors:** James C. Barton, Jackson C. Barton, Luigi F. Bertoli

**Affiliations:** 1 Department of Medicine, University of Alabama at Birmingham, Birmingham, AL USA; 2 Southern Iron Disorders Center, 2022 Brookwood Medical Center Drive, Suite 626, Birmingham, AL 35243 USA; 3 Department of Medicine, Brookwood Medical Center, Birmingham, AL USA; 4 Brookwood Biomedical, Birmingham, AL USA

**Keywords:** IgG subclass deficiency, Mannose-binding lectin, Pneumonia, Respiratory tract infection, Sinusitis

## Abstract

**Background:**

Mannose-binding lectin (MBL) deficiency may increase risk of respiratory tract infection in adults unselected for IgG or IgG subclass levels. In a retrospective study, we sought to determine associations of serum MBL levels with clinical and laboratory characteristics of unrelated non-Hispanic white adults at diagnosis of IgG subclass deficiency (IgGSD). We computed the correlation of first and second MBL levels expressed as natural logarithms (ln) in a patient subgroup. We compared these characteristics of all adults with and without MBL ≤50 ng/mL: age; sex; body mass index; upper/lower respiratory tract infection; diabetes; autoimmune condition(s); atopy; other allergy; corticosteroid therapy; and subnormal serum IgG subclasses, IgA, and IgM. We performed logistic regression on MBL ≤50 ng/mL (dichotomous) using the three independent variables with the lowest values of p in univariate comparisons.

**Results:**

There were 219 patients (mean age 51 ± 13 y; 82.5% women). Thirty-six patients (16.4%) had MBL ≤50 ng/mL. Two MBL measurements were available in 14 patients. The median interval between the first and second measurements was 125 d (range 18–1031). For ln-transformed data, we observed adjusted r^2^ = 0.9675; Pearson correlation coefficient 0.9849; and *p* < 0.0001. Characteristics of patients with and without MBL ≤50 ng/mL did not differ significantly in univariate comparisons. We performed a regression on MBL ≤50 ng/mL using: subnormal IgM (*p* = 0.0565); upper respiratory tract infection (*p* = 0.1094); and body mass index (*p* = 0.1865). This regression revealed no significant associations. Conclusions: We conclude that the proportion of the present IgGSD patients with serum MBL ≤50 ng/mL is similar to that of healthy European adults. MBL ≤50 ng/mL was not significantly associated with independent variables we studied.

## Background

Mannose-binding lectin (MBL; mannan-binding protein or lectin) is involved in innate immune defense, is produced largely by hepatocytes, and is encoded by *MBL2* (chromosome 10q21.1). MBL is an oligomer that binds repeating sugar residues (especially D-mannose and *N*-acetyl glucosamine oligosaccharides) expressed on the surfaces of diverse microorganisms [1]. MBL promotes killing of microorganisms by complement activation via the lectin pathway and by opsonization [2]. Three common *MBL2* exon 1 mutations (sometimes designated as B, C, and D) encode unstable MBL polypeptides with subnormal molecular weight, blood levels, oligimerization, ligand binding, and complement activation [3, 4] and limited acute-phase responses to severe infection [5]. *MBL2* promoter region haplotypes account for variability of blood MBL levels across race/ethnicity groups unexplained by exon 1 alleles [6]. In young male Finnish military recruits with or without asthma, MBL levels below the median were associated with significantly increased odds of respiratory tract infection, after adjustment for asthma status [7].

Immunoglobulin (Ig) G (IgG), synthesized by plasma cells, is the predominant of five classes of Igs in humans. Although the four IgG subclasses share ~ 90% amino acid identity, each subclass has different antigen binding, immune complex formation, complement activation, and half-life [8]. IgG subclass deficiency (IgGSD) in adults is characterized by: levels of IgG1–3 subclasses more than two standard deviations (SD) below the population mean; frequent or severe respiratory tract infection; suboptimal IgG responses to pneumococcal polysaccharides; and increased prevalence of autoimmune conditions [9, 10]. A study of Dutch adults with recurrent respiratory tract infection revealed no significant clinical differences between subjects with or without *MBL2* genotypes that predict low MBL production after subjects with subnormal serum Ig levels or suboptimal responses to 23-valent pneumococcal polysaccharide vaccine were excluded from the analyses [11].

We sought to determine associations of serum MBL levels with other characteristics of 219 consecutive unrelated non-Hispanic white adults at diagnosis of IgGSD in a retrospective study. We computed the correlation of first and second serum mannose-binding levels expressed as natural logarithms (ln) in a patient subgroup. We compared these characteristics of all patients with and without MBL ≤50 ng/mL: age at diagnosis; sex; body mass index (BMI); upper and lower respiratory tract infection; diabetes; autoimmune condition(s); atopy; other allergy; corticosteroid therapy; and subnormal serum levels of IgG subclasses, IgA, and IgM (dichotomous variables). We also performed logistic regression on MBL ≤50 ng/mL (dichotomous). We discuss characteristics of the present cohort and compare them with those of Caucasian white adults unselected for IgGSD who were evaluated for prevalence of low MBL levels (or corresponding *MBL2* genotypes) and risk of respiratory tract infection.

## Methods

### Patient selection

We studied consecutive unrelated self-identified non-Hispanic white adults (ages ≥18 y) in a single outpatient referral practice who reported having frequent or severe upper or lower respiratory tract infection, were diagnosed to have IgGSD [9, 10, 12] before 2 October 2018, and in whom serum MBL levels were measured. Frequent infection was defined as four or more episodes per year that required antibiotic therapy. Severe infection was defined as any infection that required in-hospital treatment.

The present study design did not include reviewing medical records of or otherwise recruiting adult control subjects from general clinic populations. We excluded patients with: common variable immunodeficiency; hypogammaglobulinemia due to B-cell neoplasms, organ transplantation, immunosuppressive therapy, anti-cancer chemotherapy, or increased Ig loss; malignancy; monoclonal gammopathy; infection with parasites, *Mycobacterium* sp., human immunodeficiency virus (HIV), Epstein-Barr virus, or cytomegalovirus; allergic bronchopulmonary aspergillosis; eosinophilic granulomatosis with polyangiitis (Churg-Strauss); ataxia-telangiectasia; rare immunodeficiency syndromes associated with elevated serum IgE levels; and incomplete evaluations.

### Other conditions

BMI was computed as kg/m^2^. We classified diabetes according to the criteria of the American Diabetes Association [13].

Upper respiratory tract infection was defined as reports of sinusitis, otitis media, mastoiditis, pharyngitis, and tonsillitis. Lower respiratory tract infection was defined as reports of bronchitis, pneumonia, and bronchiectasis.

Autoimmune condition(s), atopy (allergic asthma, allergic rhinitis, and allergic dermatitis/eczema) were diagnosed by referring physicians. Other allergy manifestations reported by patients included urticaria, angioedema, or anaphylaxis that occurred in association with specific medications, foods, or environmental allergens, or without exposure to known allergens [9].

We defined a dichotomous corticosteroid therapy variable: daily oral steroids for management of autoimmune condition(s); intermittent oral or parenteral steroids, usually to relieve manifestations of infection; or topical or inhaled corticosteroids, typically used intermittently for diverse indications [9].

### Laboratory methods

Serum Ig levels were measured using standard methods (Laboratory Corporation of America, Burlington, NC, USA) before IgG replacement therapy was initiated. We defined mean ± 2 SD as reference ranges for all Ig measurements [9, 14]. Ig reference ranges are: IgG 7.0–16.0 g/L (700–1600 mg/dL); IgG1 4.2–12.9 g/L (422–1292 mg/dL); IgG2 1.2–7.5 g/L (117–747 mg/dL); IgG3 0.4–1.3 g/L (41–129 mg/dL); IgG4 0–2.9 g/L (1–291 mg/dL); IgA 700–4000 mg/L (70–400 mg/dL); and IgM 400–2300 mg/L (40–230 mg/dL) [15]. Subnormal Ig levels were defined as those below the corresponding lower reference limits. Serum MBL levels were measured using an enzyme-linked immunosorbent assay (Laboratory Corporation of America, Burlington, NC, USA). Specific levels of MBL ≤50 ng/mL were not reported.

### Statistics

The analytic data set consisted of complete observations on 219 patients with IgGSD. For some analyses, MBL levels ≤50 ng/mL and > 4000 ng/mL were imputed as 49 ng/mL and 4001 ng/mL, respectively. Descriptive data are displayed as enumerations, percentages, mean ± 1 SD, or median (range). Age, BMI, and total IgG data were normally distributed and their respective means were compared using Student’s t-test (two-tailed). Means/medians of continuous data that were not normally distributed were compared using the Mann-Whitney U test (two-tailed). Proportions were compared using Fisher’s exact test (two-tailed). For some proportions, we computed the 95% confidence interval using continuity correction.

Retrospective chart review identified 14 patients in whom MBL was measured on different days. We computed and compared mean values of the first and second measurements. Because the range of MBL levels in adults is greater than three logs [6], we used the Pearson product-moment method to compute the linear correlation of the natural logarithms (ln) of the first and second measurements.

We compared these characteristics of patients with and without MBL ≤50 ng/mL using univariate analyses: age at diagnosis; sex; BMI; upper and lower respiratory tract infection; diabetes; autoimmune condition(s); atopy; other allergy; corticosteroid therapy; and subnormal IgG1–4 subclass, IgA, and IgM levels. Because 36 patients had MBL ≤50 ng/mL, we selected the three independent variables with the lowest values of p in univariate comparisons for multivariable logistic regression on MBL ≤50 ng/mL (dichotomous), in accordance with the “one in ten rule” [16]. Analyses were performed with Excel 2000® (Microsoft Corp., Redmond, WA, USA) and GB-Stat® (v. 10.0, 2003, Dynamic Microsystems, Inc., Silver Spring, MD, USA). We defined values of *p* < 0.05 to be significant. Bonferroni corrections were applied to control the type I error rate at 0.05 for multiple univariate comparisons.

## Results

### Characteristics of 219 patients

Mean age was 51 ± 13 y (median 52; range 18–90). There were 38 men (17.4%). Mean BMI was 29.9 ± 7.0 kg/m^2^. Upper respiratory tract infection was reported by 199 patients (90.9%). Lower respiratory tract infection was reported by 188 patients (85.8%). Both upper and lower respiratory tract infection was reported by 170 patients (77.6%). Thirty-four patients (15.5%) had diabetes (type 1, *n* = 2; type 2, *n* = 32).

Autoimmune condition(s) were diagnosed in 114 patients (52.1%). The most common conditions were Hashimoto thyroiditis, rheumatoid arthritis, Sjögren syndrome, and systemic lupus erythematosus (Table 1). Twelve of 38 men (31.6%) and 102 of 181 women (56.4%) had autoimmune condition(s) (*p* = 0.1079). Atopy was diagnosed in 68 patients (31.1%). Allergic asthma, allergic rhinitis, and allergic dermatitis/eczema were diagnosed in 60 patients, 14 patients, and 4 patients, respectively (Table 1). Six patients (2.7%) had two or more conditions classified as atopy. Ninety patients (41.1%) had other allergies. Seventy-eight patients (35.6%) reported corticosteroid therapy (Table 1).

**Table 1:** Characteristics of 219 adults with IgG subclass deficiency^1^

Characteristic	Mannose-binding lectin ≤50 ng/mL (*n* = 36)	Mannose-binding lectin > 50 ng/mL (*n* = 183)	Value of p^2^
Mean age, years (SD)	54 ± 15	51 ± 14	0.2909
Male, % (n)	19.4 (7)	16.9 (31)	0.8096
Mean body mass index, kg/m^2^ (SD)	31.3 ± 7.0	29.6 ± 6.9	0.1865
Upper respiratory tract infection, % (n)^4^	83.3 (30)	92.3 (169)	0.1094
Lower respiratory tract infection, % (n)^4^	75.0 (27)	82.5 (151)	0.3487
Diabetes mellitus, % (n) ^4^	13.9 (5)	15.8 (29)	1.0000
Autoimmune condition(s), % (n)^4,5^	50.0 (18)	52.5 (96)	0.8560
Atopy, % (n)^4^	33.3 (12)	30.6 (56)	0.8440
Other allergy, % (n) ^4^	44.4 (16)	40.4 (74)	0.7124
Corticosteroid therapy, % (n)	30.5 (11)	36.6 (67)	0.5702
Mean total IgG, mg/dL (SD)^6^	805 ± 218	775 ± 207	0.4555
Subnormal IgG1, % (n)	69.4 (25)	66.7 (122)	0.8472
Subnormal IgG2, % (n)	13.9 (5)	8.7 (16)	0.3538
Subnormal IgG3, % (n)	61.1 (22)	66.7 (122)	0.5662
Subnormal IgG4, % (n)	2.8 (1)	7.7 (14)	0.4750
Subnormal IgA, % (n)	2.8 (1)	4.9 (9)	1.0000
Subnormal IgM, % (n)	22.2 (8)	10.4 (19)	0.0565

^1^Abbreviations: *SD* standard deviation. Subnormal Ig levels were defined as those > 2 SD below the respective means: IgG1 < 4.2 g/L (< 422 mg/dL); IgG2 < 1.2 g/L (< 117 mg/dL); IgG3 < 0.4 g/L (< 41 mg/dL); IgG4 0 g/L (< 1 mg/dL); IgA < 700 mg/L (< 70 mg/dL); and IgM < 400 mg/L (< 40 mg/dL) [15]

^2^Comparisons were made with Fisher’s exact test (two-tailed). These are nominal values of p. Bonferroni correction for 17 comparisons yielded a revised p for significance of < 0.0020

^3^Upper respiratory tract infection was defined as reports of sinusitis, otitis media, mastoiditis, pharyngitis, and tonsillitis. Lower respiratory tract infection was defined as reports of bronchitis, pneumonia, and bronchiectasis

^4^These conditions were diagnosed before referral for the present evaluations

^5^These autoimmune conditions (n) were diagnosed in 114 of 219 patients (52.1%): Hashimoto thyroiditis (44); rheumatoid arthritis (27); Sjögren syndrome (26); systemic lupus erythematosus (20); psoriatic arthritis (8); ankylosing spondylitis (6); Graves disease, psoriasis, and Raynaud phenomenon (5 each); inflammatory arthritis (3); autoimmune diabetes, Crohn disease, multiple sclerosis, polyarthritis with iritis/uveitis, and sarcoidosis, (2 each); and chronic inflammatory demyelinating polyneuropathy, Guillain-Barré syndrome, mixed connective tissue disorder, myasthenia gravis, polymyositis, transverse myelitis, Schnitzler syndrome, Sweet syndrome, ulcerative colitis, undifferentiated connective tissue disease, and vitiligo (1 each). Two or more autoimmune conditions were diagnosed in 46 of 219 patients (21.0%)

^6^Proportions of patients with and without MBL ≤50 ng/mL who had total IgG < 7.0 g/L (< 700 mg/dL) did not differ significantly (38.9% (*n* = 14) vs. 39.0% (*n* = 71); p = ~ 1.0000)

#### MBL levels

Mean MBL levels of patients aged ≤52 y vs. > 52 y at diagnosis did not differ significantly. Mean/median MBL levels of men were greater than those of women, but the difference was not significant (1756/1542 ng/mL vs. 1229/789 ng/mL, respectively; *p* = 0.1526, Mann-Whitney U test). Respective mean MBL levels of patients with BMI < 30.0 kg/m^2^ and those with BMI ≥30.0 kg/m^2^ did not differ significantly. There was a weak negative correlation of ln MBL levels and BMI (adjusted r^2^ = 0.0218; Pearson correlation coefficient − 0.1478; *p* = 0.0288).

Mean/median MBL levels of patients who did and did not report upper respiratory tract infection did not differ significantly. Mean/median MBL levels of patients who did and did not report lower respiratory tract infection did not differ significantly. Respective mean/median MBL levels of patients with and without diabetes, autoimmune conditions, atopy, other allergies, and corticosteroid therapy did not differ significantly.

#### First and second MBL levels in 14 patients

**Fig. 1:**
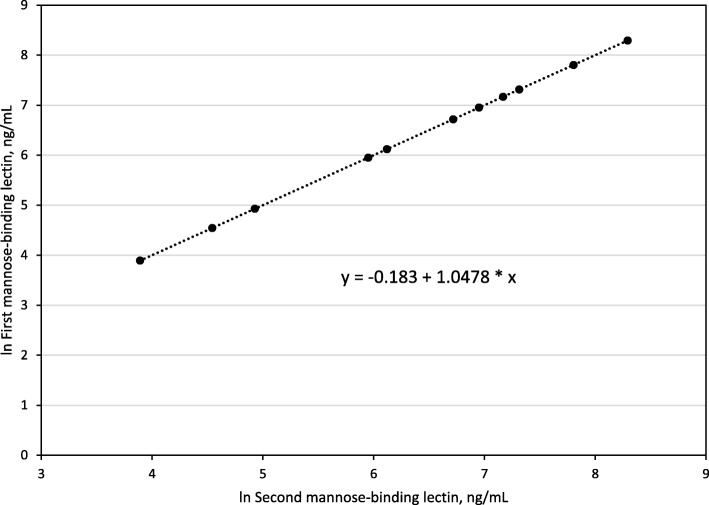
First and second serum mannose-binding levels in 14 adults with IgG subclass deficiency. Correlation of first and second serum mannose-binding levels expressed as natural logarithms (ln) in 14 adults with IgG subclass deficiency. The median interval between the first and second measurements was 125 days (range 18–1031). The lowest point represents observations on four patients. For ln-transformed data: adjusted r^2^ = 0.9675; Pearson correlation coefficient 0.9849; *p* < 0.0001. For non-transformed data: adjusted r^2^ = 0.7933; Pearson correlation coefficient 0.8935; *p* < 0.0001 (graph not shown)

The median interval between the first and second MBL measurements was 125 days (range 18–1031). Mean/median first and second MBL levels (1063/446 ng/mL and 885/420 ng/mL, respectively) did not differ significantly. There was a highly significant positive linear correlation of first and second MBL levels (Fig. 1).

#### Patients with and without MBL ≤50 ng/mL

**Fig. 2:**
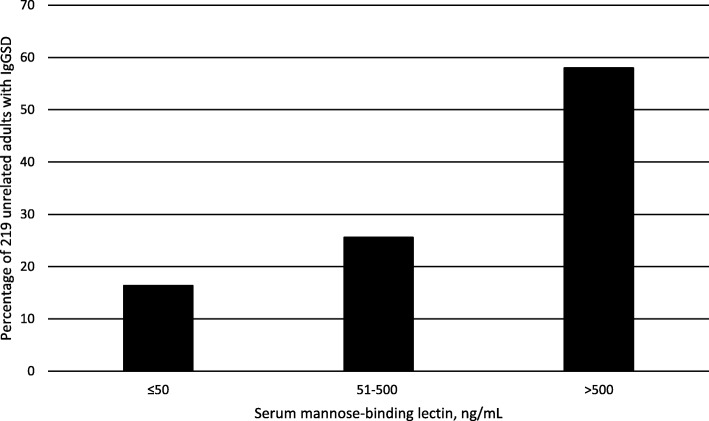
Serum mannose-binding lectin levels in 219 adults with IgG subclass deficiency

Thirty-six of 219 patients (16.4% [95% confidence interval 11.9, 22.2]) had MBL ≤50 ng/mL (Fig. 2). Characteristics of patients with and without MBL ≤50 ng/mL did not differ significantly in univariate comparisons (Table 1). Proportions of IgG subclass immunophenotypes in patients with and without MBL ≤50 ng/mL did not differ significantly (Table 2).

**Table 2:** Subnormal serum IgG subclass levels in 219 adults with IgG subclass deficiency

Subnormal IgG subclass immunophenotypes^a^	Mannose-binding lectin ≤50 ng/mL (*n* = 36)	Mannose-binding lectin > 50 ng/mL (*n* = 183)	Value of p^2^
IgG1 alone, % (n)	38.9 (14)	29.5 (54)	0.3245
IgG2 alone, % (n)	0	0.55 (1)	1.0000
IgG3 alone, % (n)	25.0 (9)	29.0 (53)	0.6906
IgG1/IgG3, % (n)	22.2 (8)	25.7 (47)	0.8338
IgG1/IgG4, % (n)	0	3.3 (6)	0.5925
IgG2/IgG3, % (n)	8.3 (3)	1.1 (2)	0.0324
IgG3/IgG4, % (n)	0	2.2 (4)	1.0000
IgG1/IgG2/IgG3, % (n)	2.8 (1)	6.6 (12)	0.6992
IgG1/IgG3/IgG4, % (n)	2.8 (1)	1.6 (3)	0.5151
IgG2/IgG3/IgG4, % (n)	0	0.55 (1)	1.0000

^a^Subnormal IgG subclass levels were defined as those > 2 standard deviations below the respective means: IgG1 < 4.2 g/L (< 422 mg/dL); IgG2 < 1.2 g/L (< 117 mg/dL); IgG3 < 0.4 g/L (< 41 mg/dL); and IgG4 0 g/L (< 1 mg/dL) [15]

^2^Comparisons were made with Fisher’s exact test (two-tailed). These are nominal values of p. Bonferroni correction for 10 comparisons yielded a revised p for significance of < 0.0050

#### Logistic regression on MBL ≤50 ng/mL

We performed a regression on MBL ≤50 ng/mL using the three independent variables with the lowest values of p in univariate comparisons: subnormal IgM (*p* = 0.0565); upper respiratory tract infection (*p* = 0.1094); and body mass index (*p* = 0.1865) (Table 1). This regression revealed no significant independent associations (*p* = 0.7197, subnormal IgM; *p* = 0.0679, upper respiratory tract infection; and *p* = 0.1268, body mass index) (*p* = 0.1734 for significance of the model).

## Discussion

Serum MBL ≤50 ng/mL occurred in 16.4% of the present 219 patients. Twelve of 100 healthy Danish blood donors (12.0%) had plasma MBL < 50 ng/mL [17]. Twenty-seven of 164 healthy white Spanish adults (16.5%) had *MBL2* genotypes that predicted low MBL levels (mean serum MBL 62 ± 9 (SD) ng/mL) [18]. Thus, the proportion of the present patients with MBL ≤50 ng/mL is similar to proportions of control adults in two healthy western European cohorts with low MBL levels or *MBL2* genotypes that predict low MBL levels. First and second MBL levels in 14 of the present patients were similar, consistent with a report that variation of MBL levels in healthy adults over time is not significant [19]. General clinical and laboratory characteristics of the present patients with MBL ≤50 ng/mL and those with MBL > 50 ng/mL did not differ significantly.

MBL ≤50 ng/mL was not significantly associated with age at IgGSD diagnosis in the present patients (mean age 51 y). In common variable immunodeficiency, the mean age of disease onset was significantly lower in patients with *MBL2* exon 1 mutations and promoter haplotypes that predict low MBL production than in patients with wild-type *MBL2* alleles (15 y vs. 25 y, respectively) [20].

MBL levels and BMI were inversely associated in a correlation analysis, although this association was not especially strong. Mean MBL levels of the present patients with BMI < 30.0 kg/m^2^ and those with BMI ≥30.0 kg/m^2^ did not differ significantly. BMI was not a significant predictor of MBL ≤50 ng/mL in a logistic regression. In a study of German adults not selected for IgGSD or frequent or severe respiratory tract infection, mean plasma MBL levels in severely obese and healthy, lean subjects did not differ significantly [21]. In obese German and Swedish subjects, MBL levels did not change significantly after weight loss [21, 22]. MBL mRNA is not expressed in adipose tissue [22].

Mean/median MBL levels of the present men and women did not differ significantly. In healthy American military beneficiaries (83% Caucasians), the prevalence of serum MBL < 50 ng/mL was significantly lower in men than women (14% vs. 20%, respectively) and the prevalence of MBL > 2000 ng/mL was significantly higher in men than women (28% vs. 21%, respectively) [23]. In a review of 84 studies, Falagas et al. concluded that males in all age groups develop respiratory tract infections (except sinusitis, otitis externa, and probably tonsillitis) more frequently than females, and that differences in sites of respiratory tract infection between males and females are probably influenced by diverse genetic, biochemical, anatomical, and socioeconomic factors [24].

The prevalence of upper and lower respiratory tract infection was lower in the present patients with MBL ≤50 ng/mL than in patients with MBL > 50 ng/mL, but these differences were not statistically significant. In British adults with chronic obstructive pulmonary disease, those with *MBL2* genotypes that predicted low MBL levels had lower risk of infective exacerbations, greater lung microbiota diversity, and decreased airway inflammation [25]. In adults with rheumatoid arthritis, estimated rates of verified upper respiratory tract infections per 100 patient-years did not differ significantly between those with serum MBL < 100 ng/mL and those with higher MBL levels [26].

Lower respiratory tract infection was reported by 86% of the present patients with IgGSD, but we observed no significant relationships of MBL and lower respiratory tract infection. The frequencies of *MBL2* alleles and genotypes in Spanish white adults with community-acquired pneumonia and control subjects did not differ significantly, but *MBL2* genotypes that predicted low MBL levels were associated with significantly greater severity of illness and risk of death [18]. In a case-control study of Spanish adults, there was no significant relationship of *MBL2* genotypes and pneumococcal community-acquired pneumonia (with or without bacteremia) [27]. In British adults with chronic obstructive pulmonary disease, the risk of infective exacerbations was significantly lower in patients with *MBL2* genotypes that predicted low MBL levels [25]. In Norwegian adults hospitalized for treatment of community-acquired pneumonia, 13% had serum MBL < 50 ng/mL and 34% had subnormal serum Ig levels, but MBL < 50 ng/mL was not associated with specific causative microorganisms, severity of illness, or short- or long-term outcomes [28]. Taken together, these reports suggest that associations of low MBL levels and lower respiratory tract infection in cohorts of Caucasian adults unselected for subnormal total IgG or IgG subclasses vary according to specific infective microorganisms, the presence or absence of underlying lung disease, and serum Ig levels.

Mean MBL levels of the present patients with and without diabetes did not differ significantly. MBL ≤50 ng/mL (dichotomous) was associated with decreased odds of diabetes in a logistic regression. In a multivariable regression on MBL levels (continuous), there was a positive association with diabetes. Mean serum MBL levels were significantly higher in Danish adults with type 1 diabetes and normal urine albumin levels than in healthy age- and sex-matched control subjects [29]. Mean serum MBL levels in Danish adults with type 2 diabetes and healthy blood donors did not differ significantly [30]. Frequencies of *MBL2* genotypes were similar in Swedish adults with type 2 diabetes [31] and subjects in the general European population [17, 32].

Autoimmune condition(s), an aggregate variable in this study, was not significantly associated with MBL ≤50 ng/mL or MBL levels. In Czech adults with autoimmune thyroid disease and healthy Czech control subjects, the prevalence of *MBL2* polymorphisms did not differ significantly [33]. In Danish adults with rheumatoid arthritis, the prevalence of undetectable serum MBL was significantly higher than that of control subjects (11% vs. 3%, respectively) [34]. Among Australian adults with rheumatoid arthritis, serum MBL < 56 ng/mL was associated with a significantly increased risk of infection that required in-hospital management [35]. In Finnish patients with primary Sjögren syndrome and control subjects, there was no significant difference in *MBL2* genotype or allele frequencies [36]. In Dutch adults with systemic lupus erythematosus (77% Caucasian), functional MBL activity < 10% of normal was not associated with increased infection risk [37]. In contrast, homozygosity for *MBL2* alleles that predicted low MBL levels was higher in Danish patients with systemic lupus erythematosus than in control subjects, and was associated with significantly greater infection risk [38].

Atopy, defined as a dichotomous variable in this study, was not significantly associated with MBL ≤50 ng/mL or MBL levels. In Finnish adults with asthma and control subjects, there was no significant association of *MBL2* genotype and atopy (defined as a wheal reaction to one or more of 22 allergens applied by skin pricks) [39].

MBL ≤50 ng/mL was not significantly associated with subnormal IgG subclasses, IgA, or IgM. In another study, five of 12 American adults with chronic rhinosinusitis and MBL < 100 ng/mL also had subnormal IgG subclass levels, including three with subnormal IgG3 [40]. In contrast, MBL < 20 ng/mL in Finnish adults with IgA < 0.05 g/L, alone or in combination with subnormal IgG subclass levels, was not associated with increased risk of respiratory infection [41].

Decreased IgG responses to 23-valent pneumococcal polysaccharide vaccination are common in adults with IgGSD [10, 12, 42, 43]. In Dutch adults with recurrent respiratory tract infections who were unselected for total IgG or IgG subclass levels, responses to 23-valent pneumococcal polysaccharide vaccination were similar in subjects with or without functional MBL activity < 10% of normal [11].

A strength of this study is that this sample size provides statistical power to estimate independent joint effects in multivariable models. Limitations of this study include lack of: control subjects; identification of microorganisms associated with infections; *MBL2* genotyping; evaluation of lectin and opsonization pathways; and evaluation of 23-valent pneumococcal vaccination responses. Family studies and longitudinal follow-up of the present patients were beyond the scope of this study.

## Conclusions

We conclude that the proportion of the present IgGSD patients with serum MBL ≤50 ng/mL is similar to that of healthy European adults. MBL ≤50 ng/mL was not significantly associated with independent variables we studied.

## Abbreviations

BMI: Body mass index
Ig: Immunoglobulin
IgGSD: IgG subclass deficiency
MBL: Mannose-binding lectin
SD: Standard deviation
